# Modeling the Costs of Postpartum Dysgalactia Syndrome and Locomotory Disorders on Sow Productivity and Replacement

**DOI:** 10.3389/fvets.2017.00181

**Published:** 2017-10-30

**Authors:** Jarkko K. Niemi, Paula Bergman, Sami Ovaska, Marja-Liisa Sevón-Aimonen, Mari Heinonen

**Affiliations:** ^1^Economics and Society, Natural Resources Institute Finland (Luke), Seinäjoki, Finland; ^2^Faculty of Veterinary Medicine, University of Helsinki, Helsinki, Finland; ^3^Economics and Society, Natural Resources Institute Finland (Luke), Helsinki, Finland; ^4^Green Technology, Natural Resources Institute Finland (Luke), Jokioinen, Finland; ^5^Faculty of Veterinary Medicine, Department of Production Animal Medicine, University of Helsinki, Helsinki, Finland

**Keywords:** dynamic programming, economic loss, sows, post partum dysgalactia syndrome, locomotory disease, longevity, piglet mortality, litter size

## Abstract

Postpartum dysgalactia syndrome (PPDS) and locomotory disorders are common health problems in sows. Previous research suggests that they can cause substantial losses, reduce sow welfare, and result in premature removal of the sow from the herd. However, economic consequences of PPDS and locomotory disorders have not been investigated thoroughly. The goal of this study was to examine economic losses caused by PPDS and locomotory disorders and their impacts on sow longevity. A stochastic dynamic programming model, which maximizes return on sow space unit and assesses sow replacement under several scenarios, was developed. The state variables were litter size, parity number, and sow’s health status. The model describes changes in the production parameters such as the number of piglets born and piglet mortality. Herd data originating from commercial sow herds and from a research farm were used to parameterize the model. Sow longevity, health, and economic results are related to each other. Eliminating the risk of PPDS from the model increased the value of sow space unit by €279 when compared to the baseline scenario. Eliminating the risk of locomotory disorders increased value by €110. Results suggest that these estimates correspond to about €29.1 and €11.5 in economic costs per housed sow during her lifetime. The estimated magnitude of losses was €300–€470 per affected sow for PPDS and €290–€330 per affected sow for locomotory disorders. However, realistically speaking, not all of these costs are avoidable. Due to premature replacement associated with these two disorders, the average number of litters that the sow would deliver during her lifetime is decreased by about 0.1–0.4 litters depending on the scenario. We also observed that the optimal lifetime of a sow is not a fixed number, but it depends on her productivity level as well as health status. In general, a healthy sow could stay in the herd until she has produced 6–10 litters. Research is needed to understand the structures and interactions underlying health impairments, performance, replacement policies, and farm economics, and to provide pork producers with management recommendations.

## Introduction

Citizens perceive animal health and welfare as important dimensions of animal production (1, 2). Also from the producers’ perspective, they are very important issues because production diseases in pigs can cause substantial losses [e.g., Ref. (3, 4)]. Diseases alter appetite, feed digestibility, impair the utilization of nutrients, and affect respiratory efficiency (5). Thereafter, health disorders typically continue to harm productivity of the affected animals in ways extending beyond the known pathological effects, potentially for a long time even after having been successfully treated.

Increased mortality and premature removal of sows from the herd have been studied widely (6–8), but relative to the overall effect of diseases in sow populations, they represent only a small proportion. At the herd level, fertility and productivity of sows as well as the quality of piglets are typically altered, and thereby, herd output and renewal potential is impaired. Consequently, involuntary herd turnover is increased, planned genetic progress deteriorated, and parity profile and overall performance of the herd adversely affected (9). Reduced sow longevity has also economic impacts (10). If an increased disease incidence leads to too high use of antimicrobial drugs, it can also be economically costly to the producer (11).

A number of studies have investigated the dynamics of sow herds, integrating directly observable, consistently and coherently reported information, by using data and records obtained for instance from farm production monitoring software (12–14). Simultaneously, economically costly outbreaks of some diseases, such as PRRS [e.g., Ref. (15, 16)] or *Actinobacillus Pleuropneumoniae* [e.g., Ref. (17)], have received attention. However, research on disorders of sows that cause less obvious losses to production and economics is scarce (18).

In this study, we focus on postpartum dysgalactia syndrome (PPDS) and locomotory disorders, which are common health problems occurring in sows. These diseases can cause productivity losses, elevated mortality, treatment costs, and premature sow removal from the herd [e.g., Ref. (19, 20)]. Furthermore, they have important welfare implications (20). However, there is limited research on the economic consequences of these two diseases in sows (4).

Postpartum dysgalactia syndrome and locomotory disorders influence sow longevity, which leads to economic losses [e.g., Ref. (21)]. For example, the costs of locomotory disorders in sows can range from a few dozens of euros up to €180 € per lame sow [e.g., Ref. (18, 22, 23)]. Wallgren et al. (22) estimated the cost of mastitis in sows in a median case at €95 per sow and substantially higher costs in the most severe cases, which were likely PPDS. Stalder et al. (24) reported that 21–35% of sows are removed from the herd due to reproductive failures, which include PPDS, and that 9–15% of sows are removed due to locomotory disorders. Regarding removal, a sow may be culled involuntarily due to the sow not recovering from a disease, or voluntarily due to poor productive performance stemming from the disease.

Because economic losses due to these two disorders are related to longevity, a well-designed sow replacement protocol is of vital importance to producers. The challenge, from the modeling perspective, is to identify important factors in the system, e.g., parity, reproductive efficiency, and frailty indicators, and incorporate them robustly in to the model. From a decision-making viewpoint, a major challenge is to account for the uncertainty and variation, especially in litter size. From several perspectives, there is a need for improved understanding of the links between animal health, productivity, sow removal, and economics.

This study contributes to the literature on the economic importance and sow removal implications of PPDS and locomotory disorders. The aims of this study are to: (1) assess economic burden of two common diseases in sows, namely PPDS and locomotory disorders and (2) examine parity and sow removal from the herd. We develop a numerical optimization model that simulates the production cycle of a sow and evaluates the replacement decision (i.e., removing a sow from the herd by culling and replacing her with a pregnant gilt).

## Materials and Methods

### Diseases Studied

Postpartum dysgalactia syndrome affects both the sow and her litter. It occurs most commonly within the first 3 days after farrowing. Insufficient milk production is the most important symptom. Mastitis with or without total agalactia, oedema of the mammary gland, vaginal discharge, coprostasis, hyperthermia, apathy, and inappetence can also be observed. Although sows often show no clear symptoms at an early stage, the disease can be diagnosed by observing the piglets; PPDS is a primary cause for neonatal problems such as diarrhea, crushing, inanition, and poor growth [see, e.g., Ref. (19, 25–28)]. The phenomenon is sometimes referred to as problem litters.

Postpartum dysgalactia syndrome is stated to be the most common disease complex of sows after parturition [e.g., Ref. (29)]. Its etiologies and signs are numerous, and the dominant representation of this disease complex varies from herd to herd. Diagnostics, register keeping, and treatment differ greatly between herds as well as between reported studies. Thus, within and between herd, prevalence estimates are problematic to compare. In Belgium, 34% of herds reported having PPDS-related problems during the previous year (30). Herd-level estimates ranged from 1.1 to 37.2% (28). Average herd level PPDS incidence is approximately 13% (28, 31–35). However, very early lactation failure may affect 100% of sows in a farrowing group.

Locomotory disorders are painful conditions that alter swine physiology and behavior. They comprise variety of conditions, such as osteochondrosis, arthrosis, arthritis, leg weaknesses, paralysis, and foot or leg injuries, infections, and fractures in sows. Locomotory problems are prevalent conditions, but their clinical definitions, stage at which they are identified, and how they are treated vary greatly. Although literature has identified risk factors for leg disorders [e.g., Ref. (36)], locomotory problems are often not recognized early enough to make a successful intervention. Specific diagnosis would often require the use of different diagnostic methods such as radiography of bones and joints or bacteriology of joint fluid.

Sows suffering from locomotory problems are prone to impaired performance: they have longer lying times and are likely to have decreased appetite compared to their sound counterparts. Several studies have reported prevalences of locomotion, leg, and claw-related problems. An average of 10% lameness prevalence has been observed; however, this varies greatly between studies and among herds within the same study (20). Locomotory disorders, like PPDS, influence sow longevity [e.g., Ref. (37)].

### Dynamic Programming Model of a Farrowing Farm

#### Objective Function

Piglet production is modeled with a stochastic dynamic programming model partly similar to the Hierarchic Markov Process model in Kristensen and Søllested (13). One benefit of dynamic programming is that it can take into account the value of information when it arrives, and its impacts on decisions. The assumed objective of a farrowing farm is to maximize net returns to a sow space unit by optimizing the replacement decision (the removal of a sow by culling, followed by the purchase of pregnant gilt). Sow space unit refers to the housing capacity that a sow requires during the production cycle.

The model accounts for the most important events in the productive life of a sow and its piglets. Replacement decisions are solved, and corresponding returns simulated, by state of nature, which represents observable characteristics of a sow. Parity number, piglet yield, and occurrence of a disease are used as the state variables. Uncertainty about sow performance is taken into account because exact piglet yield in the future is unknown when the producer decides on removal. By contrast, the mean and variance of biological parameters such as piglet yield are assumed to be known. The model optimizes the replacement decision on the condition that sufficient production capacity is allocated to each production stage. Hence, this maximized variable is return on investment given a specific production technology. Cost of capacity (i.e., fixed costs) is included in the model as a time-constant factor. Fixed costs are needed to make different production stages consistent, but they do not impact the optimal timing of replacement.

The Bellman equation (38) for this problem is of the form:

(1) $$\begin{array}{l} V_{t}(x_{t})=\underset{u_{t}}{\max}\left\{R_{{\;}_{t,\text{sow}}}^{\;}(x_{t},u_{t})+\beta E(V_{t+1}(x_{t+1}))\right\},\;t=1,\ldots ,\infty \text{ and where}\\ \;x_{t}^{\;}=\{x_{t,\text{prices}}^{\;},x_{t,\text{disease}}^{\;},x_{t,\text{parity}}^{\;},x_{t,\text{litter}}^{\;}\}\\ \text{ subject to: }x_{t,\text{disease}}^{\;}=\{x_{t,\text{PPDS}}^{\;},x_{t,\text{leg}}^{\;},x_{t,\text{other}}^{\;}\}\\ \;={\mathrm{Pr}}_{\text{disease}}(x_{t,\text{yield}}^{\;},x_{t,\text{parity}}^{\;})\\ \;x_{t+1,\text{litter}}^{\;}=g(x_{t,\text{parity}}^{\;},x_{t,\text{litter}}^{\;},u_{t},\varepsilon_{y})\\ \;x_{t+1,\text{parity}}^{\;}=q(x_{t,\text{disease}}^{\;},x_{t,\text{parity}}^{\;},x_{t,\text{litter}}^{\;},u_{t}^{\;})\\ \;x_{t}^{\;}\text{ and }V_{\infty }(x_{\infty }^{\;})\text{ are given}, \end{array}$$

where *t* is a time index measuring the number of farrowings elapsed from the start of production in the sow house*; x_t_* is the state vector where *x_t_*_,prices_, *x_t_*_,disease_, *x_t_*_,parity_, and *x_t_*_,litter_ represent state variables for time-constant market prices, currently observed disease symptoms, currently observed parity number (1 = first farrowing, 2 = second farrowing, etc.) and currently observed litter size (i.e., total number of born piglets in the current parity) in period *t*, respectively; *x_t_*_,disease_ refers to the occurrence of any of the relevant diseases (PPDS, locomotory disorders, other disorders) in the sow; *V_t_*(**x_**t**_**) is the value function (i.e., the maximized value of a capacity unit as a function of the state variable) in time period *t*; *R_t_*_,sow_ is a one-period returns function for time period *t*; *u_t_* is the control variable; β is discount factor; *E*(.) is an expectations operator applied on the term inside brackets; *V_t_*_+1_(**x_**t**_**_+1_) is a value function at period *t* + 1; *g* is a transition equation governing the evolution of piglet yield over time as a function of state variables and control policy; *q* is a transition equation governing the evolution of parity number as a function of other state variables and control policy; *x_t_*_,PPDS_ refers to the sow suffering from PPDS; *x_t_*_,leg_ refers to the sow suffering from locomotory disorders; *x_t_*_,other_ refers to the sow suffering from disease any other than PPDS or locomotory disorders;^1^ Pr_disease_ is the probability of observing a disease in a sow during the current parity; and *ε*_y_ is a parameter indicating variation related to change in the litter size between successive parities.

**Figure 1:**
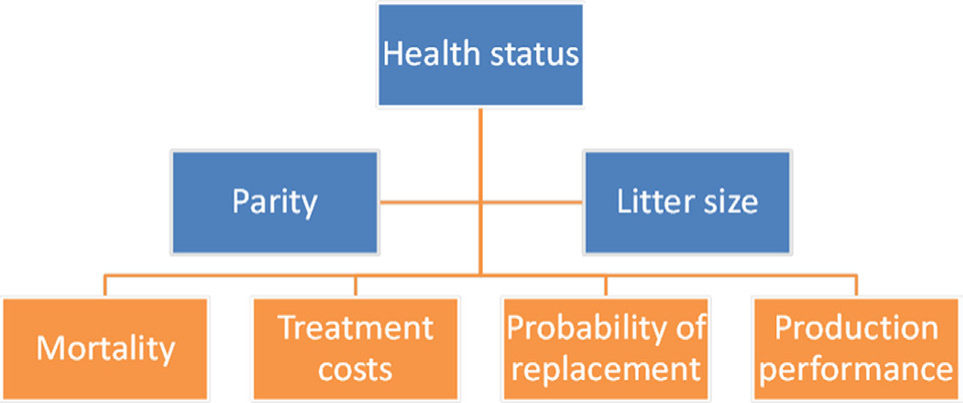
State variables (in blue) influence economic and physical performance parameters (in orange) of a sow in the dynamic programming model.

**Figure 2:**
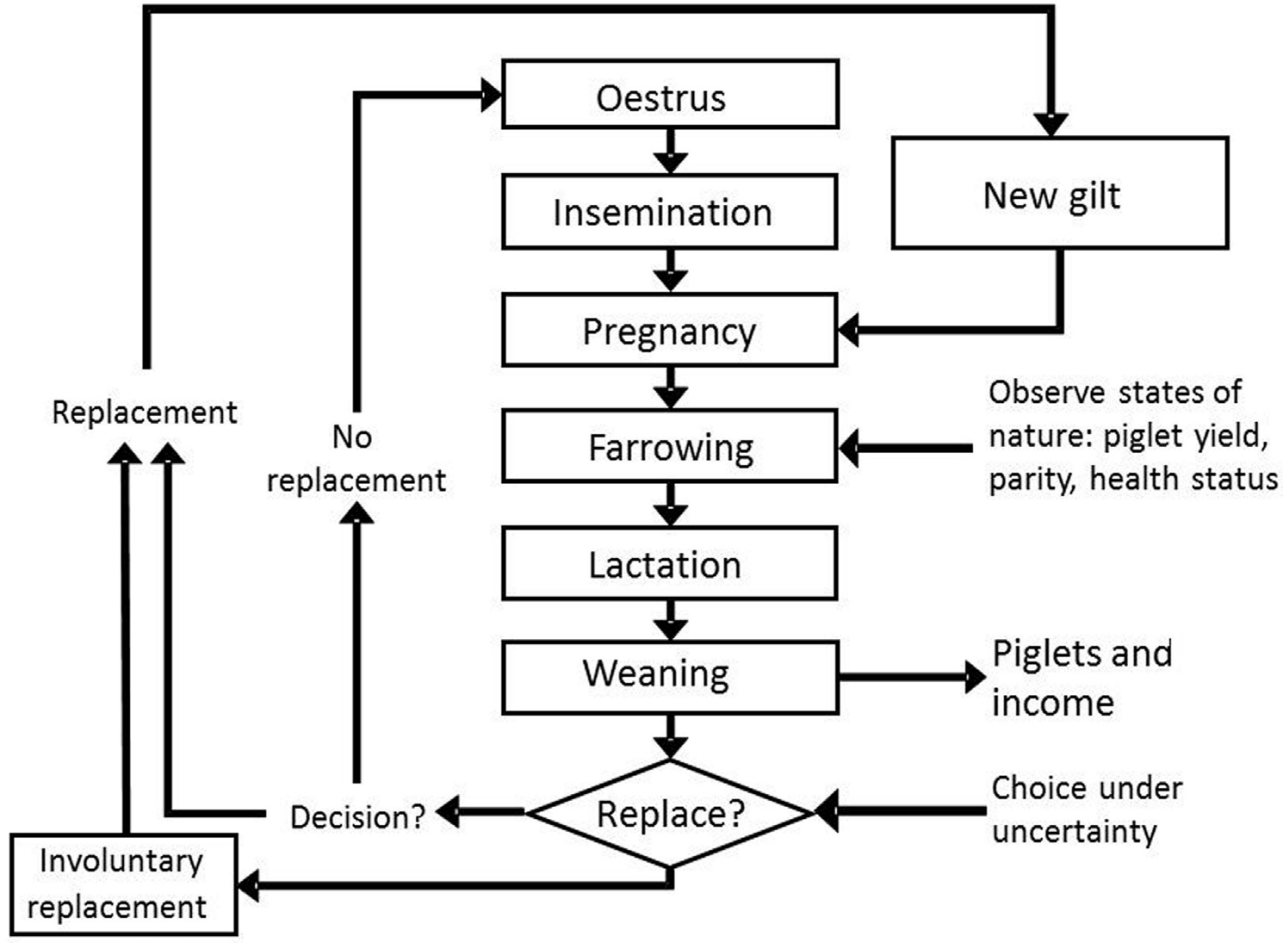
The production cycle of a sow and cash flows (revenues, costs) associated with these events, as simulated in the dynamic programming model.

The equations used in the model are specified in detail below. Transition equations for litter size and parity number have a controllable part that depends on the control variable, autonomous part that is realized deterministically, and a random part that is exogenous. In the model, we characterize how major events (Figures 1 and 2) affect cash flows, costs or revenues, during a farrowing cycle, and thus produce information needed to estimate one-period cash flows.

#### Control Variable and Parity Transition Equation

The control variable *u*_t_ can take on one of two values {0,1}, where 0 refers to not replacing the current sow after the current parity, and 1 refers to replacing the animal with a pregnant gilt. Hence, the endogenous replacement *u* is applicable to cases where exogenous replacement will not take place. Exogeneous replacement occurs with probability Pr_cull_, which is a function of parity, litter size, and sow’s health status.

The transition equation for parity number is presented below in Eq. 2. In cases where the sow is not replaced, the parity number increases by one between successive farrowings. In cases where the sow dies or is culled due to disease, poor performance, or other reason, the parity number after the removal is set at one because the replacement animal is a gilt:

(2) xt+1,parity =q(xt,disease ,xt,parity ,xt,litter ,ut ​)={xt,parity +11  if sow is not removed,  if sow is removed

The probability of removing the sow for exogenous reason, Pr_cull_, is parameterized as follows:

(3) $$\begin{array}{l} {\mathrm{Pr}}_{\text{cull}}(x_{t,\text{disease}}^{\;},x_{t,\text{parity}}^{\;},x_{t,\text{litter}}^{\;})=\mathrm{Probit}\left(\begin{array}{l} -0.608-0.041(x_{t,\text{parity}}^{\;}|1)-0.111x_{t,\text{parity}}^{\;}+0.020x_{t,\text{parity}}^{2}\\ -0.045\min(x_{t,\text{parity}}^{\;}-x_{t,\text{parity}}^{*},5)\\ +0.005\min(x_{t,\text{parity}}^{\;}-x_{t,\text{parity}}^{*},5){\;}^{2}\\ +1.214\mathrm{SBM}(x_{t,\text{disease}}^{\;},x_{t,\text{parity}}^{\;},x_{t,\text{litter}}^{\;})\\ +0.360\mathrm{SBM}(x_{t,\text{disease}}^{\;},x_{t,\text{parity}}^{\;},x_{t,\text{litter}}^{}){\;}^{2}\\ +0.980\mathrm{PM}(x_{t,\text{disease}}^{\;},x_{t,\text{parity}}^{\;},x_{t,\text{litter}}^{\;})\\ +0.520\mathrm{PM}(x_{t,\text{disease}}^{\;},x_{t,\text{parity}}^{\;},x_{t,\text{litter}}^{\;}){\;}^{2} \end{array}\right)\\ \text{ ​}-\text{0.370}x_{t,\text{disease}}^{\;}+Z(x_{t,\text{parity}}^{\;}). \end{array}$$

where SBM refers to the proportion (%) of sow’s born piglets in parity *x_t_*_,parity_ that die neonatal or perinatal; PM is the proportion of born piglets that die between farrowing and weaning. Operator “min” selects the smallest element inside the parenthesis. We restrict this element to a maximum of five, and the variable $x_{t,\text{parity}}^{*}$ refers to the number of piglets born to an average sow in the herd in a given parity (more specifically, when no “sample selection” of sows would have occurred due to removing poorly performing sows in the previous parities). Therefore, $x_{t,\text{parity}}^{\;}-x_{t,\text{parity}}^{*}$ refers to how much a sow’s current (observed) litter size deviates from the expected litter size of an average sow that has farrowed a given number of times. *Z*(*x_t_*_,parity_) is a calibration parameter that ensures the best fit for the combination of the two datasets used. For the first three parities, it has an average value of 0.12; thereafter decreasing by about 0.05 per parity. Pr_cull_ is restricted to have a minimum value of zero and a maximum value of one.

#### Data Sources

The Probit model we use is originate from Niemi et al. (39), which is based on a dataset obtained from the Finnish Animal Breeding Association (Faba), a former animal breeding cooperative in Finland. This source provided animal-level data on the productivity and genetic background of sows from 31,949 litters born in 2002. Pr_cull_ was reported for an animal of average genetic merit that was housed by a farm with the parameters similar to the sample average. Hence, our model focuses on a typical sow on a typical herd.

Beyond the Probit within Eq. 3, model was calibrated using our data collected from commercial sow herds by University of Helsinki coauthors. These data originated from 40 herds and covered 18,753 sows in the herds in 2014. We used these data to calibrate litter sizes and primiparous sow removal rate to 2014 levels. Therefore, the parameter values in our entire model are based on joint information from two datasets.

These datasets did not provide sufficient animal-level follow-up information on PPDS and locomotory disorders over several parities. Therefore, we obtained information on how the health of a sow impacts sow removal and litter size from the herd database of a former pig research station, which had been operated by Natural Resources Institute Finland (Luke) at Hyvinkää, Finland. The Luke dataset comprises 871 sows born between 1998 and 2012, and measures 2,568 litters. For each sow, the data included performance data (litter size, number of litter, etc.) and records of production diseases, veterinary and medical treatments, and exit dates with removal destination and general reasons. The effect of health disorders on productivity at various parities was obtained from the data including sows treated or not treated with antimicrobials, pain killers, or both. The data included no cases of observed production disease without treatment. For each sow, the time of entering the herd, the time of removal, and the time spent in the herd, were considered. Parameter −0.370 in Eq. 3 above is an adjustment factor that quantifies how disease in the sow contributed to removal.

#### Transition Equation for Litter Size

**Figure 3:**
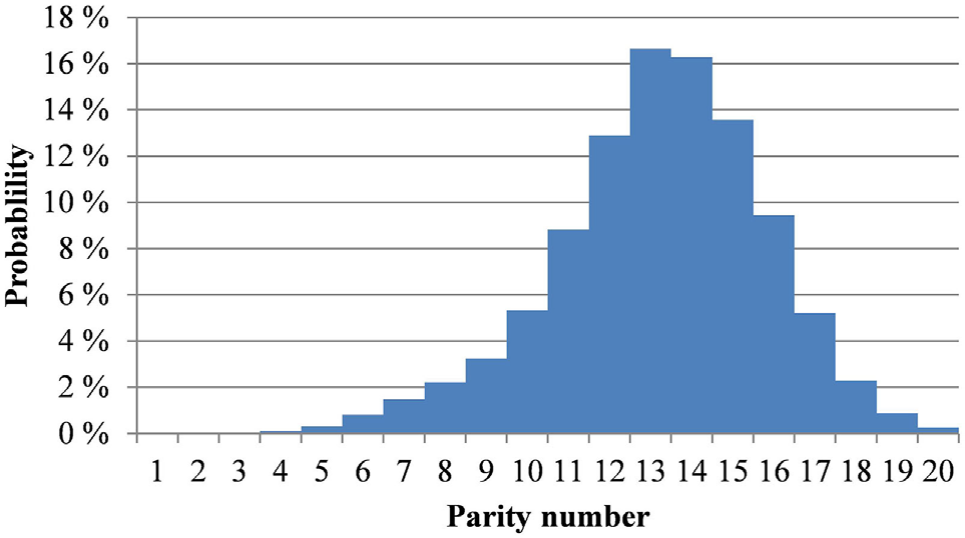
Probability distribution of litter size assumed in the dynamic programming model for primiparous sows.

Litter size (i.e., the total number of piglets born, either alive or stillborn) is a random variable whose evolution over successive parities is modeled as a stochastic process based on the commercial herds datasets:

(4) $$\begin{array}{l} x_{t+1,\text{litter}}^{\;}=\{\begin{array}{ll} \begin{array}{l} -\text{6.118}+\text{0.219}x_{t,\text{litter}}^{\;}+\text{0.700}x_{t,\text{parity}}^{\;}-0.100x_{t,parity}^{2}-\\ 0.765\text{ SBM}+0.308\text{PMT}-0.781N_{t,\text{litter}}^{\;}+\varepsilon_{\text{litter}} \end{array} & \text{if }x_{t+1,\text{litter}}^{\;}>1\\ 12.37+\varepsilon_{\text{primiparous}} & \text{if }x_{t+1,\text{litter}}^{\;}=1 \end{array}, \end{array}$$

where PMT is the ratio of piglets born alive to the total number of piglets born; *N_t_*_,litter_ is a factor used to adjust expected litter size to the currently prevailing level; *ε*_litter_ is unexplained variation in litter size (mean = 0, SD = 3.025 piglets) for multiparous sows, and *ε*_primiparous_ is variation in litter size (mean = 0, SD = 2.606) for primiparous sows. The factor 0.219*x_t_*_,litter_ refers to the repeatability of deviation of litter size when compared to the expected litter size had sample selection (i.e., removal of less productive sows) not occurred. In addition, 1.41% mortality for sows per farrowing cycle is assumed. The probability distribution for litter size of primiparous sows given in Figure 3 is based on the commercial herds datasets.

#### Hierarchical Modeling of Diseases in Pigs

Interactions between disease incidence and litter size and piglet mortality are modeled hierarchically based on the Luke data. For a given current litter size, the likelihood of a sow suffering from a disease is first adjusted according to Eqs 5–7 below. That is, the model first determines litter size, and then which individuals are suffering from disease. Next, piglet mortality (Eq. 8) and sow removal rates (Eqs 2 and 3) are determined as a function of litter size and disease. Hence, disease influences litter size through model dynamics presented in Eqs 2–4.

#### Culling Rates for Treated and Untreated Sows

To parametrize the impacts of PPDS and locomotory disorders in sows, we use the Luke data as described in more detail here. The dataset provides detailed health records for each sow. During parities 1–4, the most common reasons for treating a sow were PPDS^2^ (26–40% of treatments) and locomotory disorders (23–31%). During parities 5–9, locomotory disorders were the most common reasons for treatment (32–100%). Percentage of sows treated was highest at the first parity (18%), decreasing to 13–16% at parities 2–5, and to 11% or less at parities 6–11.

In the first parity, sows with reported disease and treatment farrowed fewer piglets compared to healthy and thus untreated sows (Table 1). However, at parities 2–4, no major differences were found. During parities 1–5, treated sows had 0.1–0.6 more stillborn piglets than untreated sows. After the fifth parity, treated sows produced fewer piglets than untreated sows.

**Table 1:** The number of liveborn and stillborn piglets (mean) by parity for sows treated healthy and thus untreated.

Parity number	Number of sows		Liveborn		Stillborn	
	Untreated	Treated	Untreated	Treated	Untreated	Treated
1	645	140	10.2	8.5	1.0	1.3
2	514	74	10.6	11.1	0.9	1.2
3	373	70	11.6	11.5	1.1	1.5
4	230	38	12.4	12.8	1.1	1.2
5	159	24	12.4	11.9	1.3	2.0
6	118	8	12.3	10.5	1.6	1.3
7	78	10	12.4	10.9	1.5	2.1
8	43	4	12.0	13.3	2.3	1.0
9	17	2	11.8	11.0	1.3	1.5
10	7	0	12.3	n/a	1.7	n/a
11	1	2	13.0	12.0	2.0	4.5

*Data are from a research at Hyvinkää, Finland*.

In the first parity, the number of piglets born alive was statistically different for treated versus untreated sows at the risk level of 0.01 (*t*-test, *p* = 0.000). However, the difference in number of stillborn piglets at the first parity was less evident (*p* = 0.070). During parities 2–4, no statistical significances were found at the 0.05 risk level. At parity 5 or thereafter, there were too few sows to permit statistical analysis.

After the first parity, 25% of sows in this dataset were removed from the herd (Table 2). Of these primiparous sows, 43% were reported to have been suffered from a production disease and been treated. As parity number increased, the percentage of removal increased and the percentage of treated sows among them decreased. During parities 1–5, the average number of piglets born alive was 0.9 higher (and the number of stillborn piglets was 0.3 lower) for sows remaining in production than for removed sows. Hence, piglet yield may have been a factor in research farm’s decision to replace a sow. Treatment rate did not seem to increase as parity number increased. On the contrary, the highest *overall* percentage of treated sows and removal rate after treatment was found at the first parity.

**Table 2:** Shares of removed and remaining sows in a research farm herd, by parity, and shares of removed sows by veterinary treatment.

Parity number	Number of litters in the data	Remaining sows, %	Removed sows, %	Removed after treatment, %	
				No	Yes
1	790	75	25	57	43
2	589	76	24	72	28
3	444	61	39	71	29
4	268	68	32	77	23
5	183	67	33	75	25
6	126	70	30	84	16
7	88	52	48	79	21
8	47	38	62	83	17
9	19	37	63	83	17
10	7	43	57	100	0
11	3	0	100	33	67

Based on the data available for each disease, the probability of occurrence for PPDS, locomotory disorder, and any other disorder is determined as a function of litter size (total number of piglets born) and parity number:

(5) Pr(xt,PPDS )=0.073−0.012xt,parity −0.047(xt,parity |=1)+ 0.056(xt,parity |≥9)+0.004xt,litter 

(6) Pr(xt,leg )=0.068−0.04xt,parity +0.085(xt,parity |≥9)−0.002xt,litter 

(7) $$\mathrm{Pr}(x_{t,\text{other}}^{\;})=0.073+0.003x_{t,\text{parity}}^{\;}+0.029(x_{t,\text{parity}}^{\;}|=1),$$

where Pr(.) refers to the probability of occurrence of the disease of interest and given the parity number and litter. Note that some parameters are multiplied by dummy variables that take a value of 1 only for given parity numbers (i.e., they are conditional).

#### Piglet Mortality

Neonatal and perinatal piglet mortality (SBM) as well as piglet mortality after birth until weaning (PM) are determined as follows:

(8) SBM=0.072+0.011ln(xt,parity)+ 0.103Pr(xt,PPDS|xt,parity =1)+ 0.019Pr(xt,PPDS|xt,parity |>1)+ 0.073Pr(xt,legs|xt,parity =1)+ 0.094Pr(xt,other),

(9) $$\text{PM}=0.096+0.018\ln(x_{t,\text{parity}}^{\;}).$$

Pr(.) is defined separately for litters of primiparous and multiparous sows as are mortality rates. The probability of locomotory disorders is defined only for the first parity because elevated mortality only occurred for the litters of primiparous sows. The probability that any other disorder is observed is defined the same for any parity. Finally, PM is defined as a proportion of the total number of piglets born (i.e., it is based on the state variable and not on piglets born alive). Parameter values for the impacts of disease on piglet mortality are calculated from the Luke’s research farm dataset as the difference between mortality rates among treated and untreated sows in the same parity. Litter-size-dependent piglet mortality is estimated using a combination of the two datasets. Piglet mortality after weaning is assumed to be fixed at 3.2%.

#### Other Physical Parameters

Other parameters are based on information collected from farms and relevant literature. Pregnancy is assumed to last for 116 days. Piglets are assumed to be weaned at the age of 28 days (9.6 kg) and sold for fattening at the age of 67 days (30 kg). A sow is assumed to return to estrus 1 week after weaning. If an insemination is unsuccessful (as 20% are assumed to be), the time interval between successive parities is increased. These sows must be re-inseminated, when they return to estrus 3 weeks later. A sow may be serviced a maximum of three times before it is removed from the herd due to infertility (7.5% of sows).

Piglets and sows were assumed to be fed according to the Finnish feeding recommendations (40). Working time needed to take care of the sows and piglets were obtained from Parviainen (41) and the space allowance (square meters) per piglet and sows was determined according to recommendations by MMM (42).

#### Price Parameters and One-Period Returns

Consistent with our model’s recursive structure, the cash flow of a piglet producing farm is described by one-period revenues and costs, which are obtained over time and separately for each time period (see Eq. 1). One-period returns depend on the state of nature, policy chosen, and economic parameters. More specifically, they take into account revenue from selling piglets, and expenses related to feeds, insemination, sow replacement, labor, and veterinary services. The total cost of producing piglets includes fixed costs, although they do not affect the model’s solution.

Table 3 describes the price parameters used in the model. These are based on national statistics and information acquired from commercial farms.

**Table 3:** Price parameters used in the dynamic programming model.

Parameter	Value	Unit
Number of piglets born per primiparous sows	13.24	Piglets/litter
Gestation feed	17.42	€/1,000 MJ NE
Lactation feed	21.19	€/1,000 MJ NE
Piglet feed	39.35	€/1,000 MJ NE
Price of labor	16.00	€/h
Price of gilt	350.00	€/gilt
Price of insemination dose	25.00	€/serving
Value of culled sow	108.00	€/sow
Price of piglet (30 kg)	55.29	€/piglet
Cost of veterinary treatment (labor, medicine)	30.00	€/treatment
Fixed housing costs	351.00	€/m^2^
Discount rate	6%	Per annum
Maintenance costs of housing	1%	Of house value
Overhead costs	4%	Per other costs

*MJ NE, mega joules net energy*.

Data regarding the cost of treatment, treatment efficacy, or the cost effectiveness are not readily available. Direct costs of treatments (veterinarian visits, procedures, and medicines) are estimated based on veterinary inspection visits, which occur six times a year, cost of medication, and increased labor. This labor is estimated as an average cost per task involved in treating an animal with either of the diseases, including veterinary care, labor to conduct the diagnosis, and administration of the treatment. Other cost consequences are determined by the equations presented in the previous sections.

A policy iteration method is used to solve the stochastic dynamic programming model [e.g., Ref. (43)]. Because litter size, piglet mortality, and parity number are stochastic factors that show covariation, Choleski factor decomposition is used when simulating these variables. Correlation between various biological parameters of the sow are based on the two datasets. Where a parameter is unavailable, we select a value based on results reported by Serenius et al. (44). The model was programmed in Matlab R2014b (8.4.0150421; The MathWorks, Inc., Natick, MA, USA).

### Scenarios

The following scenarios are simulated first and, thereafter, results are compared to the baseline scenario.

(1) The baseline scenario where the model is parametrized as described in Section “Dynamic Programming Model of a Farrowing Farm.”
(2) The incidence of PPDS is reduced by 50% from the baseline scenario.
(3) The incidence of PPDS is set at 0 (reduced by 100% from the baseline scenario).
(4) The incidence of locomotory disorders is reduced by 50% from the baseline scenario.
(5) The incidence of locomotory disorders is set at 0 (reduced by 100% from the baseline scenario).
(6) The incidence of PPDS and locomotory disorders are set at 0.
(7) The probability of removing the sow (Pr_cull_) is decreased by 0.1 (10%) from the baseline scenario.
(8) The probability of removing the sow (Pr_cull_) is increased by 0.1 (10%) from the baseline scenario.
(9) Treatment costs of sows suffering from either disease is doubled from the baseline scenario.

In addition, a sensitivity analysis on the economic impacts of diseases in response to a farm’s average sow replacement rate is conducted. This is done by simulating each scenario (1 through 9) with the probability of removal increased by reducing the calibration factor *Z*(*x_t_*_,parity_) by 0.06 points with comparison to Eq. 3. This allows us to examine the economic consequences of disorders in a herd where sow longevity is generally poorer than in the standard simulation. This may be relevant because sow replacement rates vary from herd to herd, and the initial replacement rate is expected to influence economic losses caused by various scenarios.

## Results

### Value of Sow Space Unit

**Figure 4:**
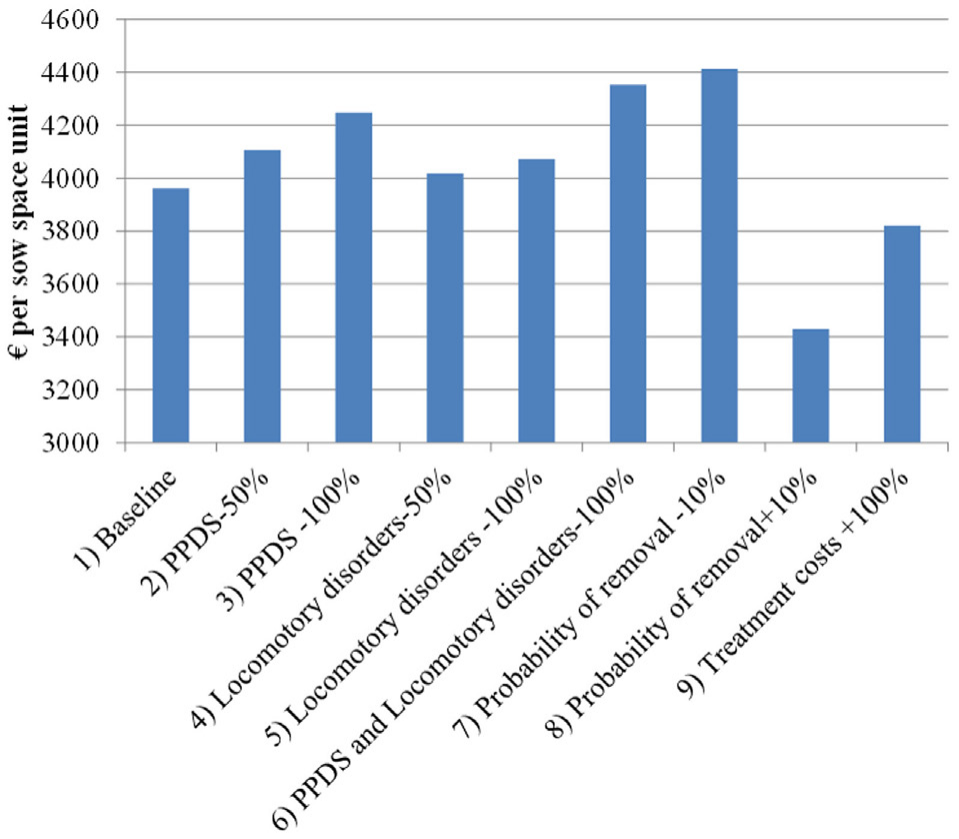
Return on fixed costs (€ per sow space unit) in the scenarios simulated by the dynamic programming model. PPDS, postpartum dysgalactia syndrome.

Figure 4 shows the return on fixed costs (or return over variable costs) for each scenario, which is measured as the value function in the first period minus the fixed costs. In the baseline scenario, it is simulated to be €3,962 per sow space unit (over entire lifetime of that unit), which on average corresponds to €12 per piglet or about €119 per litter. These estimates take into account revenues and variable costs from all sows kept at the sow space unit currently or in the future. However, after accounting for fixed costs, the simulated net present value (i.e., all discounted revenues minus all discounted costs) of the sow space unit is substantially lower. After subtracting fixed costs, the net present value in the baseline scenario falls to €313 per sow space unit.

Eliminating PPDS from the model increases the value of sow space unit by €279 (7% of baseline return on fixed cost) and eliminating locomotory disorders increases the value by €110 (3% of the baseline). This corresponds to about €29.1 and €11.5 per housed sow during her lifetime. Focusing on ill sows, PPDS is estimated to lead to losses of €300–€470 and locomotory disorders are estimated to lead to losses of €290–€330 per affected sow. Hence, for an average-sized herd in the 2014 dataset (469 sows per herd), the losses could be about €11,000 annually. Increasing the likelihood of a sow’s removal by 0.1 (+10%) decreases the value function by €546; conversely, decreasing it by 0.1 (−10%) increases the value function by €462 per sow space unit. Finally, doubling the treatment costs decreases the value function by €142 per sow space unit when compared to the baseline scenario. Doubling treatment costs for all diseases results in losses, which are close to 30% of the losses estimated to be caused by PPDS and locomotory disorders.

**Figure 5:**
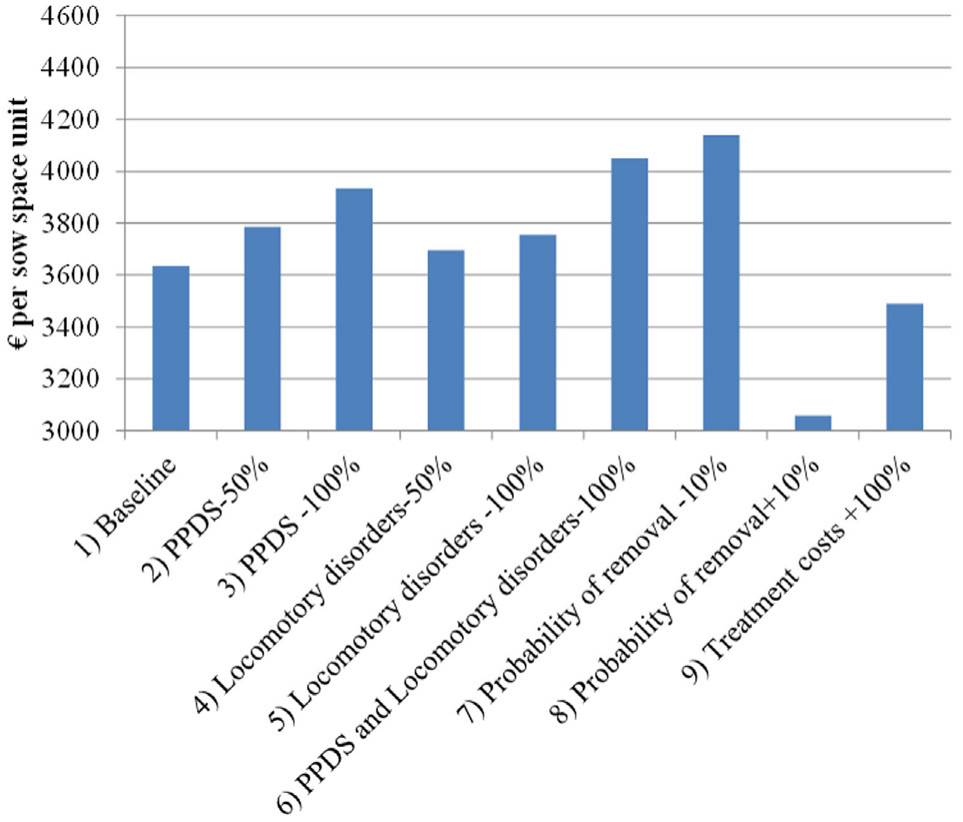
Return on fixed costs (€ per sow space unit) in the sensitivity analysis scenarios simulated by the dynamic programming model. PPDS, postpartum dysgalactia syndrome.

Recall that, for the sensitivity analysis, the model was run with the similar parameter values as above with the exception that the sow replacement rate was increased by 0.06 points by adjusting the calibration factor. With this higher probability of removal, scenarios 1 through 9 generate 2–12% larger impacts on the value function compared to the standard simulation (i.e., the magnitude of the difference between the baseline scenario and other scenarios is larger in Figure 5 than in Figure 4). For instance, the costs due to PPDS increase from €279 to €294 per sow space unit and losses due to locomotory disorders increased from €110 to €120 per sow space unit. Therefore, herds with a higher sow replacement rate appear to suffer more from diseases than herds with a lower sow replacement rate.

### Likelihood of Replacement

Table 4 summarizes, from our simulation results, the likelihood of replacement given a sow’s parity and litter size, regardless of disease status. The table shows that a sow is typically replaced after the seventh or eighth parity even if it would otherwise be in good condition. This is because the expected productivity of a primiparous sow is sufficiently high to justify the current sow’s removal. Although a sow producing smaller litters has an elevated likelihood of replacement, a smaller litter as such does not necessarily lead to removal.

**Table 4:** The likelihood of replacing a sow, by parity and litter size, as simulated by the dynamic programming model.

		Parity number								
		1	2	3	4	5	6	7	8	9
Litter size (total number of born piglets)	1	0.55	0.63	0.66	0.67	0.69	1.00	1.00	1.00	1.00
	2	0.51	0.58	0.61	0.62	0.64	1.00	1.00	1.00	1.00
	3	0.46	0.52	0.55	0.57	0.59	1.00	1.00	1.00	1.00
	4	0.42	0.48	0.50	0.52	0.54	0.56	1.00	1.00	1.00
	5	0.38	0.43	0.46	0.48	0.50	0.52	1.00	1.00	1.00
	6	0.35	0.39	0.41	0.43	0.45	0.48	1.00	1.00	1.00
	7	0.32	0.35	0.37	0.39	0.41	0.44	1.00	1.00	1.00
	8	0.29	0.32	0.34	0.36	0.38	0.41	1.00	1.00	1.00
	9	0.27	0.29	0.31	0.33	0.35	0.38	0.42	1.00	1.00
	10	0.26	0.27	0.28	0.30	0.32	0.36	0.40	1.00	1.00
	11	0.25	0.25	0.26	0.28	0.30	0.33	0.38	1.00	1.00
	12	0.24	0.24	0.24	0.26	0.28	0.32	0.37	1.00	1.00
	13	0.23	0.22	0.23	0.24	0.27	0.31	0.36	1.00	1.00
	14	0.23	0.22	0.22	0.24	0.26	0.30	0.35	1.00	1.00
	15	0.24	0.21	0.22	0.23	0.25	0.29	0.35	0.46	1.00
	16	0.24	0.21	0.21	0.23	0.25	0.29	0.35	0.46	1.00
	17	0.25	0.22	0.22	0.23	0.25	0.30	0.36	0.47	1.00
	18	0.26	0.22	0.22	0.23	0.26	0.31	0.37	0.48	1.00

**Color scale of likelihood**									

		0.20	0.30	0.40	0.50	0.60	0.70	0.80	0.90	1.00

*0 = no replacement, 1 = always replaced*.

Results from scenario 7 reveal that a 0.1 (−10%) decrease in Pr_cull_ decreases the likelihood of replacement as expected by definition, but also results in more rapid replacement of sows that produce the smallest litters, about one parity earlier than in the baseline scenario. This is because scenario 7 increases expected productivity of the subsequent sow compared to poorly yielding current sow and, therefore, makes it more profitable to replace poorly yielding sows. Moreover, increasing profitability in general shortens the production cycle in dynamic programming models. A qualitatively similar result is obtained if both PPDS and locomotory diseases are assumed to be absent. An opposite but smaller impact is obtained when Pr_cull_ is increased by 0.1 (+10%). The removal of disease results in the same incentive to remove low-yielding sows sooner.

### Piglet Yields and Longevity

Table 5 summarizes, from our simulation results, lifetime piglet yields and expected number of litters produced per sow. In the baseline scenario, on average, 9.8 piglets per litter are sold and the average number of litters a sow produces is 3.5. Eliminating either PPDS or locomotory disorders from the model (scenarios 3 or 5) yields 0.1–0.3 more litters and 1–3 more piglets sold during the sow’s lifetime. However, this result was constrained by the structure of the model. Scenario 7, in which the probability of removal was decreased by 0.1 (−10%) resulted in 1.1 litters higher lifetime production than the baseline scenario. Therefore, being able to reduce the removal rate would increase sows’ lifetime productivity more substantially because then the change in removal rate applies to all sows. The removal rate can be reduced for instance, by sound and planned culling policy, which requires an in-depth understanding of herd characteristics, applying a combination of low culling rate and rigorous monitoring and health management in early parities, or by improving breeding policies and gilt selection.

**Table 5:** Lifetime piglet yield (number of weaned piglets and sold piglets) per sow, and expected number of litters produced per sow, according to the dynamic programming model for the analyzed standard simulation scenarios and sensitivity analysis scenarios.

		Standard simulation			Sensitivity analysis		
		Piglets sold	Piglets weaned	Number of litters	Piglets sold	Piglets weaned	Number of litters
(1)	Baseline	34.0	35.1	3.48	29.1	30.1	2.90
(2)	PPDS −50%	35.4	36.6	3.62	30.2	31.2	3.00
(3)	PPDS −100%	36.8	38.1	3.76	31.4	32.4	3.10
(4)	Locomotory disorders −50%	34.6	35.8	3.54	29.6	30.6	3.00
(5)	Locomotory disorders −100%	35.2	36.4	3.61	30.1	31.1	3.00
(6)	PPDS, locomotory disorders −100%	38.1	39.3	3.88	32.5	33.5	3.30
(7)	Probability of removal − 10%	44.2	45.7	4.56	37.3	38.6	3.80
(8)	Probability of removal + 10%	26.8	27.7	2.72	23.3	24.1	2.30
(9)	Treatment costs doubled	34.1	35.2	3.49	29.1	30.1	2.90

*PPDS, postpartum dysgalactia syndrome*.

*All percentage changes in the scenarios refer to a change from the baseline scenario*.

**Figure 6:**
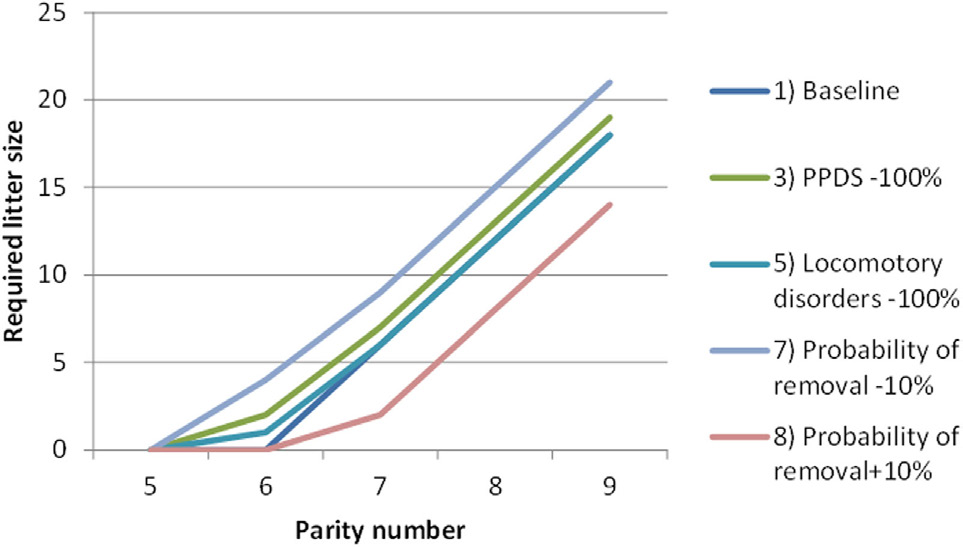
The minimum litter size (by parity number) that a sow must to exceed in order to remain in the herd, according to five scenarios simulated by the dynamic programming model. PPDS, postpartum dysgalactia syndrome.

Figure 6 illustrates, for a subset of scenarios, how large a litter would have to be in each parity for the sow to be replaced. For instance, according to the baseline scenario, a sow must produce at least six piglets in their seventh parity and at least 12 piglets in their eighth parity, or they will be replaced. However, in scenario 7, where the probability of removal is decreased by 0.1 (−10%), a larger number of piglets is required for the sow not to be replaced. In other words, although the longevity of the sow is improved, a larger litter must be produced for the sow to be kept in the herd as compared to the baseline scenario.

## Discussion

In this paper, we have examined the economic burden of two important and common disease complexes in sows, PPDS, and locomotory disorders with special attention to sow longevity. We have also examined the criteria to replace a sow by parity and litter size as identified using a numerical dynamic programming model.

We compared several scenarios associated with two health conditions. In summary, the results suggest that the losses due to the occurrence of PPDS and locomotory disorders for an average-sized herd in the 2014 dataset (469 sows per herd) could be about €11,000 annually. With 5–15% prevalence of the studied diseases in our dataset, the estimated losses per diseased sow could be €300–€470 for PPDS and €290–€330 for locomotory disorders. Further, the prevalence of these disorders as reported in the literature implies that the costs would be considerable at the country level. Expanding the perspective to national level shows that a rough estimate based on our calculations adds up to a total amount of €2 to €4 million in Finland annually. In comparison with the highly contagious diseases, which occur rarely but cause costly outbreaks (45–47), the one-time economic burden caused by PPDS and locomotory diseases is smaller. However, the constant presence and high incidence of PPDS and locomotory disorders make their overall costs likely larger than those of highly contagious diseases.

In comparison to published studies, our estimates are fairly high (4, 18, 22, 23). This may be due to several aspects, including differences in the assumed impacts of diseases on the removal rates, differences in piglet prices, and differences in the modeling approaches used.

Our results demonstrate that the optimal lifetime of a sow is not a fixed number, as Kristensen and Søllested (13) have already shown. Instead, it depends on several parameters such as litter size, piglet mortality, reproductive efficiency, and sow health. Hence, if someone suggests that it is optimal to remove a sow after a specific parity, one should explore first whether they are referring to an average optimal terminal parity. According to our results, it is optimal for a healthy sow to stay in the herd until at least sixth or seventh parity. Thereafter, it is optimal to remove the sows producing the smallest litters, but sows producing large litters might stay until their ninth or tenth parity.

When evaluating sow longevity, it is essential to pay attention to variation in parameters such as litter size, sow replacement rate, piglet mortality, and sow health. Sow productivity, especially litter size, can vary substantially from litter to litter, with important economic consequences. Yet, sows in their first three to five parities should not necessarily be removed due to a small litter size because litter size varies so much. However, some recent studies have suggested also the opposite, i.e., more intensive use of information regarding the first and second parity performance, when deciding to remove a sow [e.g., Ref. (48)]. Litter size could indeed play a more important role in sow removal if a producer is able to reduce the overall removal rates and improve sow health, because at a lower overall removal rate, the sow needs to produce larger litters to stay in the herd.

Our study design limits the generalizability of our results to individual farms. Disease-related parameter values in our model were collected from a small research farm with thorough record keeping and educated diagnostic abilities. Our results are estimated at the sow level and represent the distribution of outcomes at the herd level. Because the situation may vary from herd to herd, applying the results will require information on farm-specific factors. The information needed includes detailed data on animals and knowledge on the costs of treatment and treatment efficacy or cost-effectiveness, which are often unavailable.

The benefit of our approach, though, is that it can be used to conduct what-if-analyses. In this study, we showed that the elimination of PPDS and locomotory disorders has the potential to improve return on sow space unit and increase sow longevity. The animal disease literature has suggested potential measures to lower the incidence of these two diseases. These measures include, depending on the disease and risk factors present at the farm, gilt development, and selection, improvements in sows’ nutrition and hygiene, quality of stockmanship, and flooring (28, 49–52). Although the diseases could in theory be fully eradicated, in practice, not all costs caused by the two diseases are avoidable. Preventive measures also incur costs. Hence, the potential to improve return on sow space unit is less than the estimated economic burden of the diseases. Our results can nevertheless motivate producers to consider taking actions. Investigating the economic rationale of these measures is beyond the scope of our study, but our model could be used in such an investigation, which has not been conducted thoroughly from an economic perspective.

Production diseases of sows are a challenging area for prevention, treatment, clinical research, and modeling as they produce substantial economic burden for the whole pork production chain at all levels and impairs welfare of individual animals. Given the lack of data currently available, rigorous studies are still needed to quantify this burden appropriately to and determine the best course for accurate diagnosis, treatment, and prevention strategies. Improved understanding of the costs related to diseases can help motivate the implementation of direct animal health interventions. Improving animal health offers a win–win opportunity to improve both the farm economic performance and animal welfare.

## Author Contributions

JN was the main responsible for this study. He contributed to all parts of the study and, especially, developed the model application in practice, undertook data analysis and initial drafting of the article, and contributed to in outlining the research focus and the idea of the article. PB collected literature on diseases in sows, information based on the 40 farms dataset and on the current level of productivity of sows farms, and to the discussion section of the article. SO analyzed data from the experimental farm used in the model and provided information to the manuscript. M-LS-A contributed the data, which were used to quantify litter size, piglet mortality, and sow replacement rates without a disease, and she contributed to planning of the initial dynamic model. MH supervised the project and contributed to planning the analyses and outlining the research focus and idea of the article. All authors contributed to preparing the article.

## Conflict of Interest Statement

The authors declare that the research was conducted in the absence of any commercial or financial relationships that could be construed as a potential conflict of interest.

## Footnotes

^1^Other disorders were not modeled explicitly but they were included in the model for consistency.

^2^We considered the udder-related dysfunctions that occur during the postpartum period from day 0 to 4 following parturition.
